# Biomechanical analysis of rollator walking

**DOI:** 10.1186/1475-925X-5-2

**Published:** 2006-01-06

**Authors:** Tine Alkjær, Peter K Larsen, Gitte Pedersen, Linda H Nielsen, Erik B Simonsen

**Affiliations:** 1 Institute of Medical Anatomy, The Panum Institute, University of Copenhagen, Blegdamsvej 3C, DK-2200 Copenhagen N, Denmark

## Abstract

**Background:**

The rollator is a very popular walking aid. However, knowledge about how a rollator affects the walking patterns is limited. Thus, the purpose of the study was to investigate the biomechanical effects of walking with and without a rollator on the walking pattern in healthy subjects.

**Methods:**

The walking pattern during walking with and without rollator was analyzed using a three-dimensional inverse dynamics method. Sagittal joint dynamics and kinematics of the ankle, knee and hip were calculated. In addition, hip joint dynamics and kinematics in the frontal plane were calculated. Seven healthy women participated in the study.

**Results:**

The hip was more flexed while the knee and ankle joints were less flexed/dorsiflexed during rollator walking. The ROM of the ankle and knee joints was reduced during rollator-walking. Rollator-walking caused a reduction in the knee extensor moment by 50% when compared to normal walking. The ankle plantarflexor and hip abductor moments were smaller when walking with a rollator. In contrast, the angular impulse of the hip extensors was significantly increased during rollator-walking.

**Conclusion:**

Walking with a rollator unloaded the ankle and especially the knee extensors, increased the hip flexion and thus the contribution of hip extensors to produce movement. Thus, rollator walking did not result in an overall unloading of the muscles and joints of the lower extremities. However, the long-term effect of rollator walking is unknown and further investigation in this field is needed.

## Background

**Figure 1:**
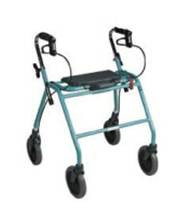
This rollator, a Dolmite Maxi 650, Dolomite AB, Anderstorp, Sweden, was used in the study. It resembles a typical rollator with four wheels, handles with brakes and a seat.

The rollator is a popular assistive walking device in most European and especially the Nordic countries [1]. The exact number of rollator users is unknown but about 6.4% of Danish 56–84 year-old people use a rollator and in Sweden about 4% of the total population use a rollator [1]. The terms "wheeled walker", "rolling walker", "three-wheeled walker", four-wheeled walker" [2-4] are frequently used synonymously with rollator, which can be defined as a frame with three or four wheels; the rollator has handles with brakes, and in some cases it has a seat, a basket or a tray (Fig. 1) [1].

The main purpose of using a rollator is to improve the walking performance and minimize the risk of falling. Studies have shown that the walking performance in elderly subjects measured in terms of distance, cadence and velocity is improved when they walk with a rollator [2]. Furthermore, a recent study has shown that rollator users are generally satisfied with their rollator and consider it an important prerequisite for living a socially active and independent life [1].

However, knowledge about how the rollator affects the walking pattern is limited. To our knowledge no studies have investigated the biomechanical differences between walking with and without a rollator except from one study that observed a reduction in the vertical ground reaction force during rollator walking [5]. Such information may be clinically relevant in the decision-making process of whether a rollator would be beneficial to a subject or not, or whether the use of a rollator should be supplemented with e.g. balance and/or strength training. Studies of walking with canes or walking poles have shown that these walking-aids reduce the load on the lower extremities [6-8]. Presumably, the rollator reduces the loads on the leg muscles and the joints to some extent as well. However, the specific changes in kinematic and kinetic walking pattern parameters when walking with a rollator have not yet been quantified.

It is unclear whether an unloading of certain muscle groups and joints during walking would impair the functional ability during other types of daily physical activities and movements like sit-to-stand, short walking distances, stair climbing, balance control during standing/squatting etc. One study concluded that the use of walking-aids combined with a high activity level may protect against falls in elderly subjects [9]. Thus, information about how muscle groups and joints in the lower extremities are affected by walking with a rollator may be used in the development of specific rehabilitation strategies in elderly and disabled rollator users.

Accordingly, the purpose of the present study was to investigate the biomechanical effects of walking with a rollator on the walking pattern of healthy subjects. The reason for studying a group of healthy subjects was that it was both unethical and difficult to ask actual rollator users to walk without their rollator.

## Methods

### Subjects

Seven healthy women (age: 34.7 (range: 25–57) years, height: 1.70 (range: 1.64–1.78) m, weight: 64.7 (range: 55–75) kg) participated in the study. None of the subjects had any history of injuries or musculo-skeletal dysfunctions in their lower extremities. All subjects gave their informed consent to participate in the experiments which were approved by the local ethics committee.

### Gait analysis

The subjects were fitted with fifteen small reflecting spherical markers (12-mm diameter) according to the marker set-up described by Vaughan et al. [10]. The markers were placed on the head of the fifth metatarsal, the heel, the lateral malleous, the tibial tubercle, the lateral femoral epicondyle, the greater trochanter, the anterior superior iliac spine and sacrum. All subjects wore lightweight flexible shoes with a thin, flat sole. The subjects were asked to walk across two force platforms (AMTI, OR6-5-1) both with and without a rollator (Fig. 1, Dolmite Maxi 650, Dolomite AB, Anderstorp, Sweden) at a speed of 4.5 km/h. The rollator was adjusted to each subject in an upright standing position with the arms hanging down along the body so that the handles were on a level with processus styloideus ulnae. The Dolmite Maxi 650 rollator model was used because it was wide enough to pass next to the force platforms without touching them. However, pilot studies showed that the wheels of the rollator sometimes hit the first platform anyway. To solve this problem a metal rail was fixed to the ground along the first platform to ensure that the rollator wheels did not touch it.

The subjects were allowed to practice walking both with and without the rollator to become familiar with the movements and the pre-determined walking speed. The speed was controlled by photocells, which made it possible to teach the subjects to approach 4.5 km/h.

Five video cameras (Panasonic WV-GL350) operating at 50 Hz were used to record the movements. The video signals and the force plate signals were synchronized electronically with a custom-built device. The device put a visual marker on one video field from all cameras and at the same time triggered the analogue-to-digital converter which sampled the force plate signals at 1000 Hz. The subjects triggered the data sampling and synchronization when they passed the first photocell.

The video sequences were digitized and stored on a PC. Sixteen non-coplanar points on a standard calibration frame (Peak Performance 5) were digitized to calibrate each of the video sequences. The calibration frame was placed in the middle of the walkway and covered both force plates. Three-dimensional co-ordinates were then reconstructed by direct linear transformation using the Ariel Performance Analysis System (APAS).

Prior to the calculations, the position data were digitally low-pass filtered by a fourth order Butterworth filter with a cut-off frequency of 6 Hz, and the 1000 Hz force plate signals were downsampled to 50 Hz to fit the video signals.

### Calculations

Internal flexor and extensor joint moments about the ankle, knee and hip were calculated using a three-dimensional inverse dynamics approach described by Vaughan et al. [10]. Furthermore, the internal adductor/abductor moment was calculated for the hip joint. The joint moments were expressed in an anatomically based reference system. The anatomical axes for the flexor and extensor moments of the ankle, knee and hip joint were the mediolateral axes of the segment reference frames of the shank, the thigh and the pelvis, respectively [10]. The anatomical axis for the hip adductor/abductor moment was the so-called floating axis that was perpendicular to the mediolateral axis of the pelvis segment frame and the longitudinal axis of the thigh segment frame [10]. Ankle dorsiflexor, knee extensor, hip flexor and hip abductor joint moments were considered positive, while ankle plantarflexor, knee flexor, hip extensor and hip adductor joint moments were considered negative. The angular impulse (i.e. the area under the joint moment curve) quantifies the total contribution of a joint moment towards producing movement. It has been shown that in some cases the angular impulse values may be relevant to the evaluation of walking patterns of different groups [11]. Accordingly, the angular impulse (Nm· s) was calculated by integration of the area under the joint moment curves. The angular impulses of the plantarflexors (i.e. the negative part of the ankle moment), knee extensors (i.e. the first positive part of the knee moment) and the hip extensors (i.e. the negative part of the sagittal hip moment curve), flexors (i.e. the positive part of the sagittal hip moment curve) and abductors (i.e. the positive part of the frontal hip moment curve).

The peak values as well as the angular impulses of the ankle, knee and hip moment were calculated and used as input parameters for the statistical analyses.

The angular position of the ankle, knee and hip joints was calculated to describe the movements in the sagittal plane. In addition, the angular position of the hip movement in the frontal plane was calculated. Zero degrees defined the anatomical position (foot at 90° to leg) and positive values reflected ankle dorsiflexion, knee hyperextension, hip flexion and hip abduction.

The average angle as well as the range of motion (ROM) [12], i.e. the difference between the maximum and minimum joint angles, were calculated for the stance phase and used as input parameters for the statistical analyses.

MATLAB was used for all calculations.

### Data reduction

Data obtained from the left leg during the stance phase were analyzed. Six gait cycles were normalized and averaged for each subject and situation (with (rollator-walking) and without (normal walking) a rollator, respectively). Normalization was performed by interpolating data points to form 500 samples for each gait cycle. The joint moments were normalized to body mass. Ensemble averages were then calculated for rollator (n = 7) and normal walking (n = 7) using the mean value for each individual subject.

### Statistics

A Student's t-test for paired data was used to identify statistically significant differences between rollator- and normal walking in selected kinematic and kinetic variables of the walking patterns. All results are presented as means (SD). The level of significance was set at 5%.

## Results

There was no difference in the walking speed between rollator- (4.49 (0.05) km/h) and normal walking (4.51 (0.03) km/h) (p = 0.731).

**Figure 2:**
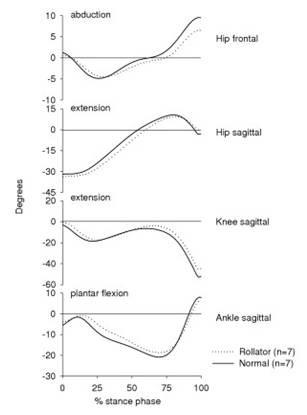
Average joint angular curves (degrees) of the ankle, knee and hip in the sagittal plane and of the hip in the frontal plane. Dotted lines reflect walking with a rollator (n = 7) and solid lines reflect normal walking (n = 7). Positive values indicate hip abduction/extension, knee extension and ankle plantarflexion. 0 % on the x-axis is heel strike and 100 % is toe-off.

The joint angular kinematics were significantly different between rollator- and normal walking (Fig. 2, Table 1). During rollator-walking the ankle and knee joints were less dorsiflexed/flexed and had a smaller ROM than during normal walking (Fig. 2, Table 1). In contrast, the hip joint was more flexed during rollator-walking than normal walking (Fig. 2, Table 1). There was no difference in the hip ROM in the sagittal plane, while the hip ROM in the frontal plane was significantly smaller during rollator-walking than normal walking (Table 1).

**Table 1:** Joint position variables.

**Variable**	**Rollator (n = 7)**	**Normal (n = 7)**	***P*-value**
**Ankle – sagittal**			
Avg stance pos	-9.0 (3.4)	-10.5 (2.3)	0.031
ROM in stance	25.2 (2.8)	29.0 (3.1)	0.031
**Knee – sagittal**			
Avg stance pos	-13.0 (3.9)	-15.9 (4.0)	0.013
ROM in stance	44.8 (3.6)	49.5 (1.9)	0.013
**Hip – sagittal**			
Avg stance pos	-10.6 (4.6)	-8.0 (3.0)	0.013
ROM in stance	43.9 (2.5)	42.8 (2.7)	0.166
**Hip – frontal**			
Avg stance pos	-0.7 (3.1)	0.2 (2.0)	0.211
ROM in stance	11.6 (3.7)	14.5 (2.3)	0.029

Values are means (SD) in degrees. Negative values indicate flexion/dorsiflexion/adduction. Avg stance pos, average angular position during stance. ROM, range of motion.

**Figure 3:**
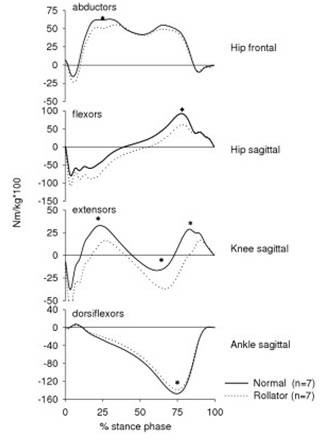
Average joint moment curves (Nm/kg·100) of the ankle, knee and hip in the sagittal plane and of the hip in the frontal plane. Dotted lines reflect walking with a rollator (n = 7) and solid lines reflect normal walking (n = 7). Positive values indicate hip abductor/flexor dominance, knee extensor and ankle dorsiflexor dominance. 0 % on the x-axis is heel strike and 100 % is toe-off. Asterisks indicate statistical significant differences between peak values of the joint moments during rollator- and normal walking.

The joint moments were significantly different at each joint between the two situations (Fig. 3, Table 2). The peak plantarflexor moment and the plantarflexor angular impulse of the ankle joint were significantly smaller during rollator-walking than during normal walking (Fig. 3, Table 2). The knee joint moment was significantly reduced during rollator-walking (Fig. 3, Table 2). During rollator-walking both the peak knee joint moments and the angular impulse of the knee extensors were reduced by approximately 50% when compared to normal walking (Fig. 3, Table 2). The angular impulse of the hip flexors was significantly smaller during rollator-walking (Fig. 3, Table 2). In contrast, the angular impulse of the hip extensors was significantly larger during rollator-walking than during normal walking (Fig. 3, Table 2). Thus, the shift from hip extensor dominance to flexor dominance in the stance phase occurred significantly later during rollator-walking (54.5% (9.5) % of stance phase) than during normal walking (40.0% (7.7) % of stance phase) (p < 0.001) (Fig. 3).

**Table 2:** Joint moment variables.

**Variable**	**Rollator (n = 7)**	**Normal (n = 7)**	***P*-value**
**Ankle – sagittal**			
Peak plantar flexor	-138.9 (4.9)	-147.9 (5.2)	0.020
Plnt flex ang imp	-24.8 (5.6)	-28.5 (5.6)	0.007
**Knee – sagittal**			
Peak knee extensor*	16.4 (19.7)	33.7 (14.5)	0.010
Peak knee flexor	-37.4 (17.0)	-17.4 (14.7)	0.001
Peak knee extensor**	18.6 (4.0)	31.0 (11.9)	0.010
Knee ext ang imp	1.9 (2.0)	3.8 (2.0)	0.000
**Hip – sagittal**			
Peak hip extensor	-118.3 (20.6)	-99.6 (25.6)	0.197
Peak hip flexor	64.0 (12.8)	92.7 (16.9)	0.000
Hip ext ang imp	-13.3 (3.2)	-8.1 (2.7)	0.000
Hip flex amg imp	7.3 (2.5)	12.1 (3.2)	0.000
**Hip – frontal**			
Peak hip abductor*	58.2 (8.3)	66.0 (7.1)	0.017
Peak hip abductor**	51.1 (14.7)	56.3 (8.8)	0.114
Hip abd ang imp	21.6 (5.5)	24.0 (2.7)	0.085

Values are means (SD). Peak moments are expressed as Nm/kg·100. Plantar flexor angular impulse (Plnt. flex. ang. imp), knee extensor angular impulse in the first half of stance (Knee ext. ang. imp.), hip extensor/flexor angular impulses (Hip ext./flex. ang. imp.) are expressed as Nm·s. *Peak moment in the first half of the stance phase. ** Peak moment in the second half of the stance phase.

The peak hip abductor moment in the first half of the stance phase was significantly smaller during rollator-walking than during normal walking (Fig. 3, Table 2). Although the angular impulse of the hip abductors tended to be smaller during rollator-walking no statistical significance was observed in this parameter between the two situations (Table 2).

## Discussion

The present study demonstrated significant differences between normal and rollator-walking patterns. The study included seven healthy subjects between the ages of 25 and 57 years who were able to walk with and without a rollator at identical walking speeds (4.5 km/h), which is important when comparing joint moment curves [13-15].

The main findings of the present study showed that walking with a rollator resulted in a remarkable reduction in the knee extensor moment and thus an unloading of the quadriceps muscle during the stance phase. There were also small but significant reductions of the ankle plantarflexor and hip abductor moments. In contrast, the angular impulse of the hip extensors and the duration of the hip extensor moment were increased during rollator-walking. The hip joint was generally more flexed throughout the whole stance phase during walking with the rollator, while the ankle and knee joint were less dorsiflexed/flexed. In addition, the ankle and knee ROM in the sagittal plane along with the hip ROM in the frontal plane were decreased during rollator-walking.

These results confirm that although the weight of the trunk was supported by the rollator, this did not result in an overall reduction of the joint moments around all three joints in the lower extremities. The unloading of the ankle and knee joints during rollator-walking seemed to be partly compensated by an increase in the hip extensor moment, which probably was needed to push the rollator in a forward direction and keep up its horizontal velocity.

The increased hip flexion throughout the whole stance phase was due to the increased forward flexion of the trunk during rollator-walking. The increased hip flexion could possibly explain the increased hip extensor moment during rollator-walking. This concurs with other studies that have observed increased hip flexion along with an increase in the hip extensor moment during walking [12].

The sagittal ankle, knee and frontal hip joint ROM's were reduced and the knee and ankle joints were less flexed during rollator-walking. During normal walking the time period between heel strike and peak knee flexion in the first half of the stance phase reflects the weight acceptance and energy absorption controlled by the knee extensors [16]. During rollator-walking the demand for knee extensor energy absorption is reduced because part of the body weight is supported by the rollator which possibly may explain the reduced knee moment and knee flexion observed in the present study. The reduced knee flexion during rollator-walking could possibly explain the reduced dorsiflexion of the ankle joint observed in this situation.

The rollator is a common and popular walking-aid among elderly and disabled subjects [1,2,17]. Rollator users are typically older than the subjects that participated in the present study or disabled and they would probably not be able to walk safely at the same walking speed without their rollator. Therefore, the observed changes in the walking pattern during walking with and without a rollator may not necessarily apply to elderly and/or disabled rollator users. However, it may be very difficult, if not impossible to investigate the differences between walking with and without a rollator in actual rollator users as they are unlikely to be able to walk without any walking-aid. Thus, in the present study a biomechanical method was established to investigate the differences between walking with and without a rollator, and the results may be used as a model for general changes in the joint moment pattern and the kinematics during rollator-walking in healthy subjects.

The rollator is definitely a very effective walking-aid that supports the body, improves the walking performance in terms of distance, cadence and velocity [2] and in many cases serves as a pre-requisite for living a normal life [1]. From a clinical viewpoint there is no doubt that if the alternative is complete immobilization of a person, the rollator seems a perfect solution that ensures at least a minimum of physical activity, which is ultimately beneficial for the cardiovascular [18,19] and the musculo-skeletal systems [20,21]. However, the rollator may also be used as part of a rehabilitation program in order to help a person to learn to walk without a walking-aid. In such situations it may be important to be aware of the results that revealed that rollator-walking led to a remarkably reduction of the knee extensor moment and thus an unloading of the quadriceps muscle, which is a very important muscle in movements like sit-to-stand, postural control, stair climbing in healthy subjects [21,22]. The hip abductor moment, which plays a significant role in balancing the trunk during walking [23], was also reduced during rollator-walking. It is unclear whether this unloading has negative consequences for balance control and functionality in other types of movement and daily activities. One study concluded that the use of walking aids combined with a high activity level may protect against falls in elderly subjects [9]. Another study concluded the functional ability was not negatively influenced in long term rollator users [24].

## Conclusion

The rollator-walking pattern in healthy subjects was characterized by increased hip flexion, decreased ankle dorsiflexion and knee flexion, and reduced the ankle and especially the knee joint moments significantly, while the contribution from the hip extensors to produce movement was increased. However, the functional consequences of these changes and the long-term effects of rollator-walking are unclear and further investigation in this field is needed.

## Authors' contributions

TA and EBS designed the study. TA, PKL, GP, LHN performed the experiments. TA was responsible for the data analysis, calculations and drafted the manuscript. PKL, GP, LHN, participated in the data treatment. EBS contributed to the discussion and the interpretation of the results. TA, PKL, GP, LHN and EBS shared the discussion. All authors read and approved the final manuscript.
